# Response of Cultured Neuronal Network Activity After High-Intensity Power Frequency Magnetic Field Exposure

**DOI:** 10.3389/fphys.2018.00189

**Published:** 2018-03-12

**Authors:** Atsushi Saito, Masayuki Takahashi, Kei Makino, Yukihisa Suzuki, Yasuhiko Jimbo, Satoshi Nakasono

**Affiliations:** ^1^Biological Environment Sector, Environmental Science Research Laboratory, Central Research Institute of Electric Power Industry, Chiba, Japan; ^2^Department of Electrical and Electronic Engineering, Graduate School of Science and Engineering, Tokyo Metropolitan University, Tokyo, Japan; ^3^Department of Precision Engineering, Graduate School of Engineering, University of Tokyo, Tokyo, Japan

**Keywords:** power frequency magnetic field, neuronal network, synchronized bursting activity, multi-electrode array, pacemaker-like neuron, inhibitory synapse

## Abstract

High-intensity and low frequency (1–100 kHz) time-varying electromagnetic fields stimulate the human body through excitation of the nervous system. In power frequency range (50/60 Hz), a frequency-dependent threshold of the external electric field-induced neuronal modulation in cultured neuronal networks was used as one of the biological indicator in international guidelines; however, the threshold of the magnetic field-induced neuronal modulation has not been elucidated. In this study, we exposed rat brain-derived neuronal networks to a high-intensity power frequency magnetic field (hPF-MF), and evaluated the modulation of synchronized bursting activity using a multi-electrode array (MEA)-based extracellular recording technique. As a result of short-term hPF-MF exposure (50–400 mT root-mean-square (rms), 50 Hz, sinusoidal wave, 6 s), the synchronized bursting activity was increased in the 400 mT-exposed group. On the other hand, no change was observed in the 50–200 mT-exposed groups. In order to clarify the mechanisms of the 400 mT hPF-MF exposure-induced neuronal response, we evaluated it after blocking inhibitory synapses using bicuculline methiodide (BMI); subsequently, increase in bursting activity was observed with BMI application, and the response of 400 mT hPF-MF exposure disappeared. Therefore, it was suggested that the response of hPF-MF exposure was involved in the inhibitory input. Next, we screened the inhibitory pacemaker-like neuronal activity which showed autonomous 4–10 Hz firing with CNQX and D-AP5 application, and it was confirmed that the activity was reduced after 400 mT hPF-MF exposure. Comparison of these experimental results with estimated values of the induced electric field (*E*-field) in the culture medium revealed that the change in synchronized bursting activity occurred over 0.3 V/m, which was equivalent to the findings of a previous study that used the external electric fields. In addition, the results suggested that the potentiation of neuronal activity after 400 mT hPF-MF exposure was related to the depression of autonomous activity of pacemaker-like neurons. Our results indicated that the synchronized bursting activity was increased by hPF-MF exposure (*E*-field: >0.3 V/m), and the response was due to reduced inhibitory pacemaker-like neuronal activity.

## Introduction

High intensity time-varying electromagnetic field (EMF) stimulates the human body through excitation of neuronal and muscle cells. The stimulus effect by the magnetic field (MF) is mainly caused by the depolarization of the voltage-gated ion channel and is detected as perception in humans and animals (Saunders and Jefferys, 2007; World Health Organization, 2007). The threshold of perception through neuronal or muscle cell excitation and the modulation of neuronal activity by MF are important biological indicators for establishing a reference value for protecting the human health from environmental EMFs. Representative international guidelines adopting the stimulus threshold as a biological basis include the International Commission on Non-Ionizing Radiation Protection (ICNIRP) guidelines (International Commission on Non-Ionizing Radiation Protection, 2010) and the Institute of Electrical and Electronics Engineers (IEEE) standards (The Institute of Electrical and Electronics Engineers, 2002). A stimulus effect called “(magneto)phosphene” is defined as one of the physiological reactions of humans with the lowest threshold in the current international guidelines or standards. Phosphene was first discovered by D'Arsonval (1896), and the phenomenon induces a flickering light during low frequency MF exposure to the retina, which is part of the central nervous system. The frequency-dependent threshold value of phosphene was evaluated precisely by Lövsund (Lövsund et al., 1979, 1980), and it became clear that the minimum threshold is around 20 Hz. In recent years, human volunteer experiments with phosphene have revalidated the threshold from 10 to 100 Hz, and it has been further investigated by the Utility Threshold Initiative Consortium (UTIC); hence, the biological threshold in humans is expected to be expanded (Souques et al., 2014).

On the other hand, in volunteer experiments with phosphene, some subjects complained of discomfort, headache, and fatigue, suggesting that MFs may have an effect on brain function (The Institute of Electrical and Electronics Engineers, 2002). These physiological responses through their influence on brain function are harmful, leading to deterioration of the quality of life; thus, MF exposure is an important biological indicator to be considered from the perspective of human body protection. Regarding the influence of low-intensity power frequency MFs on brain function, great interest has been gained and numerous findings have been obtained from various recent studies with human volunteers (Marino et al., 2004; Legros et al., 2012, 2015), experimental animals (Manikonda et al., 2007; Szemerszky et al., 2010; Balassa et al., 2013; Rauš et al., 2013, 2016; Salunke et al., 2014; Elmas and Comlekci, 2015), and cultured cells and tissues (Azanza et al., 2002, 2013; Manikonda et al., 2007; Moghadam et al., 2008, 2011; Varró et al., 2009; Moretti et al., 2013; Gramowski-Voß et al., 2015; Yang et al., 2015); however, the thresholds of neuronal modulation and implicated mechanisms by MF exposure are still under investigation. In addition, it has been recommended in the environmental health criteria (EHC) published by the World Health Organization (WHO) that the mechanism and threshold in the nervous system should be explained using cultured cells and theoretical models (World Health Organization, 2007).

The previous cellular studies that investigated the relationship between external electric field (EF) exposure and neuronal modulation have reported that the range of the threshold is approximately 0.1–5 V/m (Bawin et al., 1984, 1986; Jefferys, 1995; Francis et al., 2003; Saunders, 2003; Deans et al., 2007). Although these results contained large variations, and each threshold value was well below the threshold of the neuronal cell membrane excitation in the central nervous system [*E*-field (rms) was estimated about 8.7 V/m], and it was higher than the threshold of phosphene in the retina [*E*-field (rms) was estimated about 0.023–0.062 V/m], as estimated by theoretical models and numerical simulations (The Institute of Electrical and Electronics Engineers, 2002; Dimbylow, 2005; Hirata et al., 2011). In contrast, previous studies have evaluated the effect of MF exposure on brain function with human volunteers using functional magnetic resonance imaging (fMRI), and the results showed that human brain activity was modulated by 60 Hz, 3 mT MF exposure (Legros et al., 2015). The MF threshold of phosphene was estimated at about 24.4 mT (rms) at 60 Hz (The Institute of Electrical and Electronics Engineers, 2002); therefore, the threshold of neuronal modulation in human brain activity was suggested to be about eight times lower than that of phosphene. Based on these experimental and theoretical thresholds proposed in the previous studies, the findings of the human volunteer studies and those of the cellular studies were discordant. As for these results, it is considered that various factors, such as the method related with EF or MF exposure, differences in species, and synaptic density in cultured cells or the human brain, are involved. Therefore, in order to address these gaps in knowledge, it is necessary to develop new bottom-up approaches in cellular study to reconfirm the experimental results.

As a first step to solving these experimental problems, we focused on the relationship between EF and MF exposure. The previous experiments that used cultured neuronal networks were mainly designed for evaluating the effects of EF exposure; therefore, our present study aimed at detecting the stimulus response in cultured neuronal networks to MF exposure. To detect the stimulus response of cultured neuronal networks immediately after high-intensity power frequency MF (hPF-MF) exposure, we applied a previously developed hPF-MF exposure system combined with a multi-electrode array (MEA) system. In the present study, we report the exposure intensity-dependent response and the mechanism of neuronal modulation of the cultured neuronal network by MF exposure.

## Materials and methods

### Cell culture

**Figure 1:**
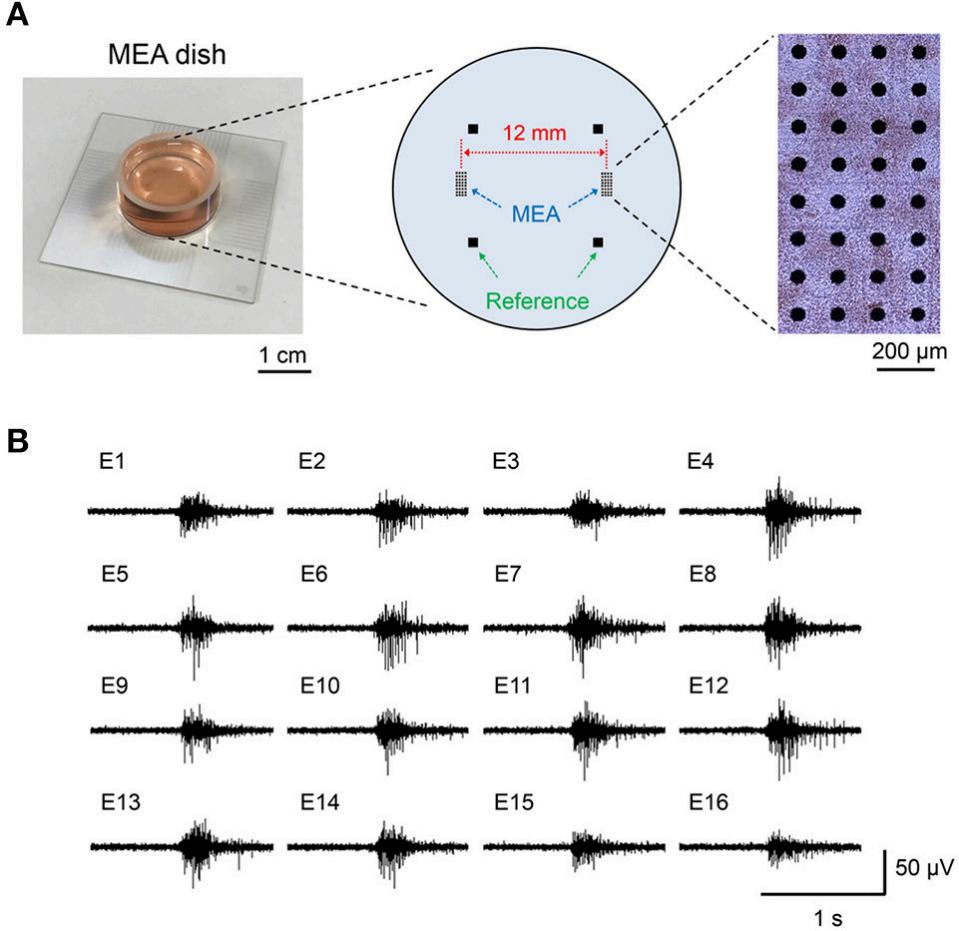
Separate-type multi-electrode array (MEA) dish and synchronized bursting activity of cultured neuronal network. **(A)** Layout of the electrodes in separate-type MEA and the photograph of the cultured neuronal network on the MEA. **(B)** An example of extracellular potential of synchronized bursting activity recorded with the MED64 system.

All animal experiments were approved by the animal experiments ethics committee at the Central Research Institute of Electric Power Industry (CRIEPI), and were performed according to the guidelines for the care and use of laboratory animals. In addition, we observed the principles of animal welfare and conducted experiments with the minimum possible number of animals. Isolation and culture of cortical and hippocampal neurons and astrocytes were performed with the following methods, based on a previous report (Saito et al., 2017). Briefly, cortical and hippocampal tissues harvested from 18- to 19-day-old Wistar rat embryos (Charles River Laboratories, Japan) were enzymatically dissociated to single cells using 0.5%, 15–20 min Trypsin solution (Sigma-Aldrich, St. Louis, MO) treatment. Dulbecco's Modified Eagle Medium (DMEM, high glucose; Thermo Fisher Scientific, Waltham, MA) containing 10% fetal bovine serum (FBS, Thermo Fisher Scientific), 5% horse serum (HS, Thermo Fisher Scientific), and 1% Penicillin-Streptomycin (Pe-St, Sigma-Aldrich) was used for the cell culture medium. To detect the neuronal network activity, we used a multi-electrode array (MEA) dish, which has 50 μm-diameter circle electrodes separated by 150 mm intervals and were arranged in 8 × 4 separated blocks (MED-P5003A; Alpha MED Scientific, Osaka, Japan) (Figure 1A). The center distance between the 8 × 4 separated blocks was 12 mm; this distance is necessary to enhance the effect of the magnetically induced current generated concentrically. The isolated neuronal and glial cells were seeded on the MEA dish with a density of 30 × 10^4^ cells and 2 mm-diameter in each separated block. During the culture, half of the medium was exchanged with fresh medium twice a week to maintain culture conditions.

### Recording of cultured neuronal network activity

#### MEA-based extracellular recording system

To evaluate the effects of MF exposure on the cultured neuronal network activity, we used a previously developed thermostatic acrylic culture chamber (TACC)-attached MEA-based extracellular recording system (Takahashi et al., 2017). The MEAs at the bottom of the culture dish permitted multi-point, wide-area, and long-term recording of spontaneous and synchronized electrical activities of cultured neuronal networks during the hPF-MF exposure experiments. The MEA dish attached to the extracellular recording system (MED64 system, Alpha MED Scientific) which was placed on the acrylic platform of the TACC. The recorded signals were amplified 10,000 times with the pre- and post-amplifiers, band passed using a 100 Hz to 2 kHz filter, and A/D converted at a 25 kHz sampling rate using the MED64 system attached software (Mobius; Alpha MED Scientific).

#### Preparation of samples

The long-term cultured neuronal networks on the MEA generate a burst-type firing, which is synchronized between a wide range of multi-electrodes (Figure 1B). The pattern of the synchronized bursting activity changes complicatedly in the developmental process of neuronal networks and it stabilizes at 1 month or more of culture (Mukai et al., 2003; Takayama et al., 2009). This synchronization has been confirmed in mammalian brains such as in rats, cats, and monkeys (Steriade et al., 1993). In addition, it was also confirmed in a human cultured neuronal network, which was differentiated from human pluripotent stem cells (Odawara et al., 2016). Therefore, we considered that synchronized bursting activity is a physiological phenomenon commonly observed in mammalian nervous systems, and it is a suitable model for functional assessment of the effects of MF exposure. In this study, the maturation of neuronal network activity was defined by the stability of the active electrodes and electrical activity patterns on the MEA dish over 30 days culture, which was based on our previous report (Saito et al., 2014).

#### Data analysis

The data recorded from the MEA-based system were analyzed by spike raster plot analysis and burst detection analysis using a MED64 system attached software (Burstscope; Alpha MED Scientific), which was based on the previously described method (Mukai et al., 2003). The number of synchronized bursting activities or single neuronal activities on the MEA dish before and after hPF-MF exposure in each exposure intensity were compared by statistical analysis using Welch's *t*-test. Here, the neuronal networks cultured on the MEA dish were exchanged in each hPF-MF exposure trial. These statistical data were expressed as mean ± standard deviation (SD). All statistically significant differences were defined as *P* < 0.05.

### High-intensity power frequency magnetic field (hPF-MF) exposure

#### hPF-MF exposure method

**Figure 2:**
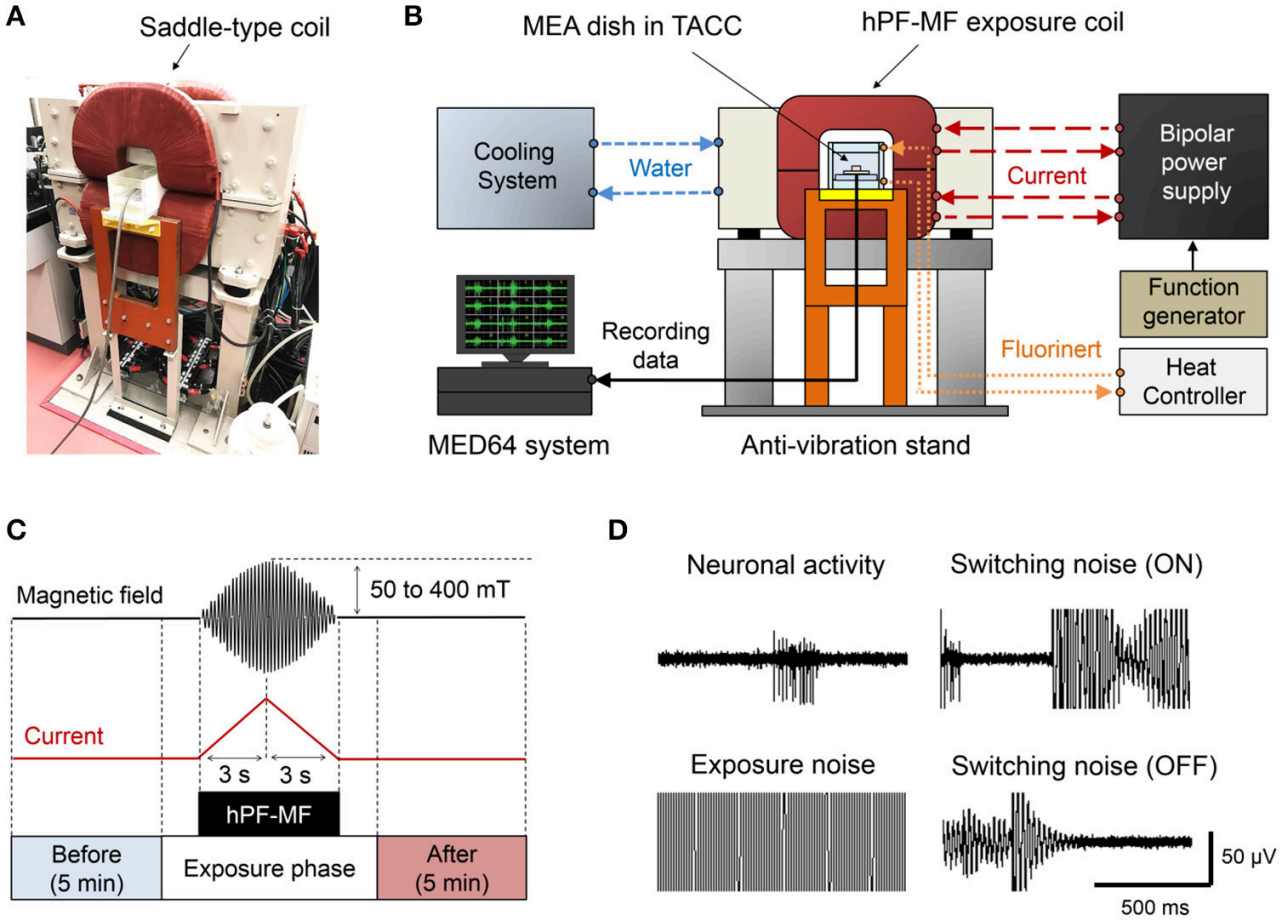
The high-intensity power frequency magnetic field (hPF-MF) exposure system. **(A)** Photograph of the saddle-type hPF-MF exposure coil. **(B)** Schematic diagram of the system. Constant temperature water is flowing into the copper wire in the coil and thermostatic acrylic culture chamber (TACC), and the temperature is 20 and 42°C, respectively. The high-speed bipolar power supply is controlled by a function generator. The MEA dish is installed in the TACC and connected to the MED64 system. **(C)** Magnetic field exposure scheme. The electrical activity of the cultured neuronal network was recorded for 5 min before and after hPF-MF exposure. To avoid mixing of artificial noise, data of a certain period before and after hPF-MF exposure were excluded. In addition, the hPF-MF was generated by a current input of a short time to the coil (rise time: 3 s, fall time: 3 s), resulting in a burst-like sweep waveform. **(D)** Example of incorporation of artifact noise. Extremely large switching noise and exposure noise superimpose on neuronal activity. Moreover, after the end of the exposure, the noise is reduced to the background level.

To modulate cultured neuronal network activity by MF exposure, we used a previously developed and customized hPF-MF exposure system (Nakasono et al., 2003; Takahashi et al., 2017). The hPF-MF exposure system consisted of a custom-made saddle-type MF generating coil (TECNO Electric Industry, Kanagawa, Japan) (Figure 2A), a custom-made high-speed bipolar power supply (NF Corporation, Kanagawa, Japan), and a function generator (WF1974; NF Corporation). The saddle-type coil included a hollow conductor (C1020T, 8.3 × 8.3 mm, φ5.2 mm) for the water-based cooling system, which contained 90 turns/coil. To generate vertically-uniformed MF, two saddle-type coils were aligned in the vertical direction at a distance of 125 mm, and combined in a window frame-type iron core. By inputting the same current to the two saddle-type coils using a high-speed bipolar power supply, the cultured neuronal network was exposed to the 50 Hz, sinusoidal, hPF-MF [magnetic flux intensity (*B*-field) was 0, 50, 100, 200, and 400 mT(rms)] during 6 s [first 3 s is 0 to *B*(max), last 3 s is *B*(max) to 0, *B*(max) is maximum *B*-field intensity]. This time duration depended on the performance of our hPF-MF exposure system to achieve minimum exposure time. The MF distribution in the vertically-uniformed MF exposure space (50 × 50 × 50 mm) was confirmed to show >95% uniformity using an exposure level tester (Model 9550; F.W. Bell, Orland, FL). The schematic view of the hPF-MF exposure system is shown in Figure 2B. Since the temperature of the coils rises when the coil is energized, heat generation was suppressed by using the circulation of constant temperature (20°C) water into the copper wire and the cage of the coil. The cultured neuronal networks were set on the MEA-based extracellular recording system that was attached to the TACC system. The TACC was connected to a circulation pump, and fluoric inactive solution (Fluorinert; 3M, Maplewood, MN) was introduced into the flow path layer provided at the top and bottom of the box. The TACC system was used to stabilize the temperature around 37°C during the experiments. In order to exclude the influence of coil vibration, the TACC was set on a base designed not to contact the MF exposure coils, and all equipment was placed on an anti-vibration stand.

#### Assessment of temperature environment during hPF-MF exposure

To evaluate the temperature during MF exposure, the temperature environment inside the cell culture medium before and after MF exposure was measured in real-time. For temperature measurement, a fiber-optic thermometer (FL-2000; Anritsu Meter, Tokyo, Japan) was used. The temperature change at the bottom of the MEA dish was measured during the hPF-MF exposure experiment. In this temperature measurement, the tip of the fiber-optic recording probe contacted the bottom of 8 × 4 electrodes for the evaluation of temperature elevation near the MEAs. In addition, when setting the MEA dish in the TACC induced changes in the temperature of the medium, we investigated the time required to stabilize its temperature elevation.

#### hPF-MF exposure during extracellular recording

The hPF-MF up to 400 mT(rms) was exposed to the cultured neuronal network during extracellular recording. Here, the amplitude of signals derived from the neuronal network activity recorded from a MEA dish was extremely weak compared to the hPF-MF exposure-related electromagnetic noise. To distinguish the noise contamination from the extracellular recording data associated with the energization of the coil, we applied short-term hPF-MF exposure and evaluated the change in neuronal network activity before and after exposure using the experimental scheme shown in Figure 2C. The characteristic of the exposure scheme is that it can expose up to 400 mT (rms), sinusoidal, short-term (rise time; 3 s, fall time; 3 s) MF to cultured neuronal networks. The recording time of neuronal activity was defined to be 5 min before and after hPF-MF exposure without noise. Using this exposure scheme, we attempted to seamlessly detect the changes in neuronal network activity immediately after hPF-MF exposure, while minimizing the mixing of electromagnetic noise. Figure 2D shows an example of the recording waveform and intensity of the extracellular potential and the electromagnetic noise observed in our experiment. Noise at the time of “ON” and “OFF” in the figure is the switching noise accompanying the energization of the coil. The amplitude of these switching noises was much larger than the amplitude of the spike waveform detected from the extracellular recording data, and we could not detect neuronal network activity during noise contamination. The background noise disappeared after shutting off the energization of the coil; the noise level was returned to the levels before hPF-MF exposure.

#### Estimation of E-fields in the MEA dish

In our experimental conditions, we used a container with uniform magnetic field and concentric circle in the vertical direction. Based on these conditions, the *E*-field in the cultured medium was calculated by the following equation: *E* = π*fBr*, where *E* is the induced EF strength, π is the circular constant, *f* is the frequency of the MF, *B* is the magnetic flux density, and *r* is the radial distance from the center of the MEA dish. From this estimation, the *E*-field induced to the neuronal networks in the culture medium was estimated.

### Pharmacological application combined experiments

#### Inhibition of inhibitory synapses

To clarify the mechanisms of the hPF-MF exposure-related stimulus response, we combined the pharmacological application for inhibition of synaptic transmission. A previous study revealed that N-methyl-D-aspartic acid (NMDA) receptors involved in excitatory synaptic transmission are essential for the generation of synchronized bursting activity in cultured neuronal networks (Nakanishi and Kukita, 1998). Therefore, we inhibited the GABA receptor associated with inhibitory synapses with 10 μM (-)-bicuculline methiodide (BMI, Sigma-Aldrich), and then we investigated the modulation of excitatory synaptic transmission after hPF-MF exposure.

#### Inhibition of excitatory synapses

A previous model-based simulation showed increase of the frequency of synchronized activity by slightly decreasing the inhibitory input (Compte et al., 2003). This hypothesis indicated that the modulation of neuronal responses via inhibitory synapses was an important indicator for the effects of MF exposure. However, in order to perform this experiment, it was necessary to select the inhibitory neurons from the cultured neuronal network that contained numerous types of neuronal cells. Here, previous studies have reported that autonomous activity of GABAergic pacemaker neurons is not blocked by the addition of excitatory synapse inhibitors D-(-)-2-amino-5-phosphonopentanoic acid (D-AP5) and 6-cyano-7-nitroquinoxaline-2,3-dione (CNQX) (Frank and Mendelowitz, 2012). In addition, it has been reported that inhibitory neurons selected by such a pharmacological method include pacemaker-like neurons autonomously firing at a frequency of 4–10 Hz (Serafin et al., 1996; Fischer et al., 1999; Varga et al., 2008). Therefore, we applied D-AP5 (Sigma-Aldrich) and CNQX (Sigma-Aldrich) to the cultured neuronal networks and evaluated the effects of hPF-MF exposure on the pacemaker-like neuronal activity.

## Results

### Evaluation of temperature in the culture medium during hPF-MF exposure

**Figure 3:**
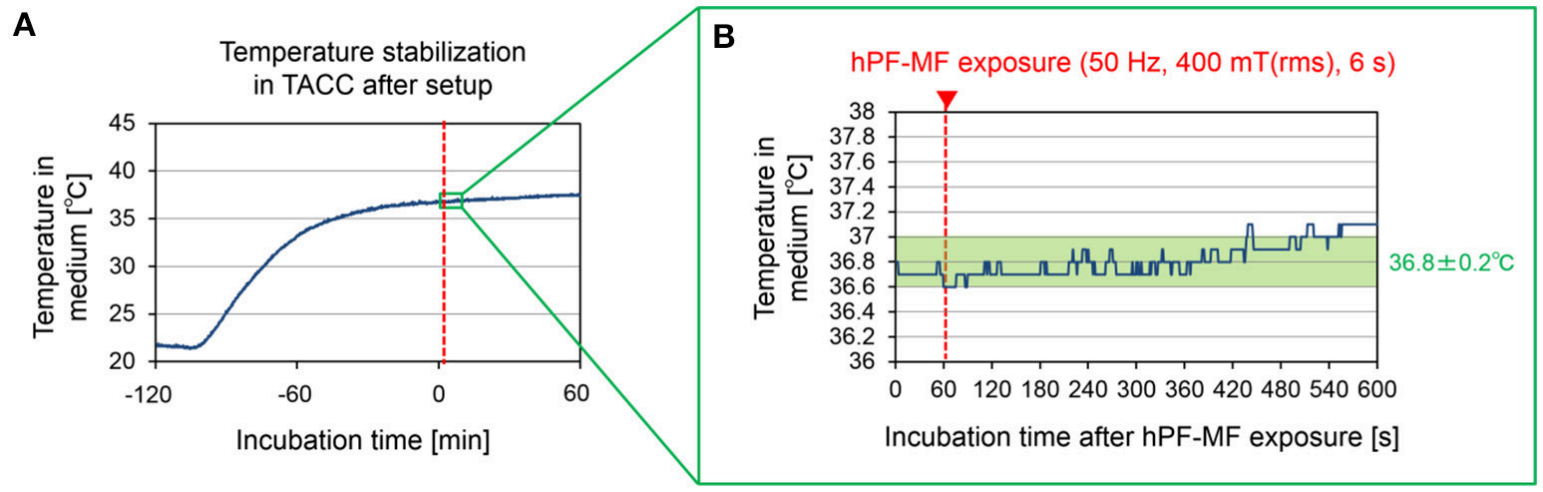
Temperature in the culture medium during the hPF-MF exposure experiment. **(A)** Temperature change in the culture medium after setting the MEAs dish to the acrylic cell culture chamber. Although the temperature inside the medium decreases with the movement and installation of the MEAs dish, the temperature is stabilized by over 2 h of culture in the chamber. **(B)** Confirmation of temperature change due to magnetic field exposure at temperature stabilization. In this experimental system, it is possible to maintain temperature fluctuation under +0.2°C during 7 min after 400 mT hPF-MF exposure. TACC: thermostatic acrylic culture chamber.

The temperature of the culture environment was stabilized around 37°C by suppressing the heat generation of the coil using the water-cooled circulation system and the TACC, according to the method shown in Figure 2B. However, as can be seen from Figure 3A, in the process of setting the MEA dish in the TACC and hPF-MF exposure coil, the medium's temperature decreased because of contact with the outside air. Therefore, in this study, cultured samples were kept for over 2 h in the TACC, and the medium's temperature was stabilized before the hPF-MF exposure experiments.

The temperature change under thermostatic conditions in the medium when the MF was 400 mT (rms), which was the maximum exposure intensity of our exposure apparatus, is shown in Figure 3B. The temperature fluctuation in the culture medium had stabilized to below 0.2°C, even at 7 min after the hPF-MF exposure. Therefore, these results indicated that the possibility of thermal influence by MF exposure can be eliminated by our hPF-MF exposure scheme and temperature control system.

### Potentiation of synchronized bursting activity by hPF-MF exposure

**Figure 4:**
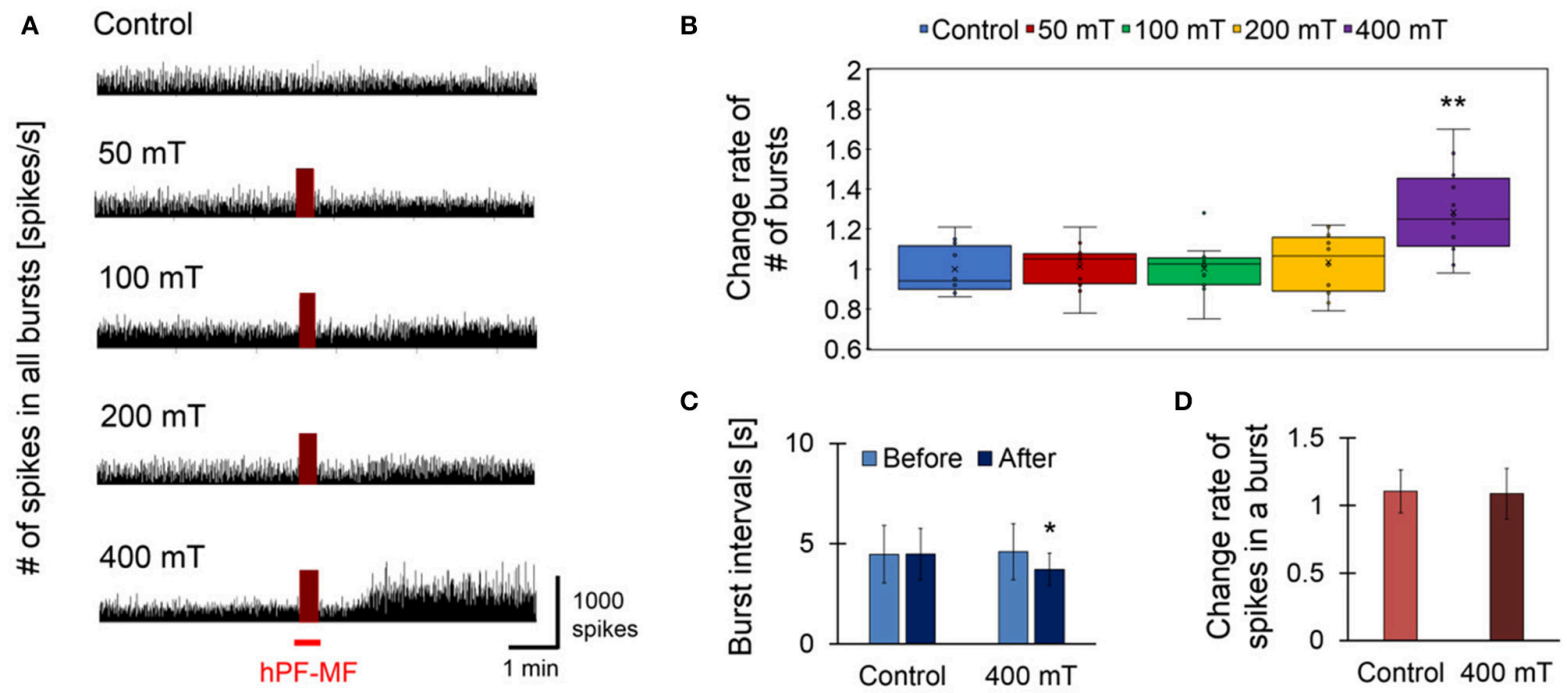
Response of cultured neuronal network due to hPF-MF exposure. **(A)** Comparison of spike firing frequency in each magnetic field exposure intensity. As a result of comparing temporal changes in the number of spikes detected from all electrodes of the MEA, an increase in spike firing frequency after exposure was observed in the 400 mT-exposed group. **(B)** Comparison of the change rate of the number of synchronized bursting activities before and after hPF-MF exposure at each intensity. In the 400 mT-exposed group, a significant increase in the synchronized bursting activity was observed compared to the Control group. **(C)** Comparison of the time interval of synchronous burst firing in the Control and 400 mT-exposed groups. In the 400 mT-exposed group, the interval of bursting activities after the 400 mT hPF-MF exposure statistically decreased compared to before the exposure. **(D)** Comparison of the number of spikes included in one bursting activity in the Control and 400 mT-exposed groups. There was no significant difference in the rate of change in spike firing frequency between the Control and 400 mT-exposed groups before and after exposure. *N* = 12 independent MEA dishes, ± SD, ^*^P < 0.05, ^**^P < 0.0001.

Figure 4 shows the results of the assessment of synchronized bursting activity before and after exposure of 50 Hz, sinusoidal short-term MF at 50, 100, 200, and 400 mT (rms). First, the comparison of the number of spikes was detected from all 64 electrodes of a MEA dish. Here, the MEA dish which exposed each hPF-MF was different, and the hPF-MF exposure in each MEA dish was not repeated per trial. The hPF-MF exposure intensity showed that the spike number clearly increased only in the 400 mT-exposed group (Figure 4A). The change rate of the number of synchronized bursting activities in the 400 mT-exposed group was 1.28 ± 0.21, which was statistically higher than that in the non-exposed (Control) group, which was 1.00 ± 0.12 (Figure 4B). In addition, significant changes in spike frequency and synchronized bursting activity were not confirmed under 50–200 mT hPF-MF exposure.

Next, we investigated the relationship between synchronized bursting intervals and spike frequency for the enhancement effect of neuronal network activity on the 400 mT-exposed group in which a clear response was observed at Figures 4A,B. Regarding the time interval of each burst firing, there was no change in the Control group from 4.47 ± 1.43 to 4.49 ± 1.27 s, whereas in the 400 mT-exposed group it was shortened from 4.60 ± 1.39 to 3.73 ± 0.808 s (Figure 4C). These results showed that the synchronized burst firing rate accelerated with the 400 mT hPF-MF exposure. However, the comparative result of the number of spikes participating in one synchronized burst firing showed that there was no increase in the number of spikes involved in synchronous burst firing (Figure 4D). Thus, these results indicated that 400 mT hPF-MF exposure increases the frequency of synchronized bursting activity at the network level without increasing the frequency of spike firings contained in a burst.

### Effect of inhibitory synapses on neuronal responses after hPF-MF exposure

**Figure 5:**
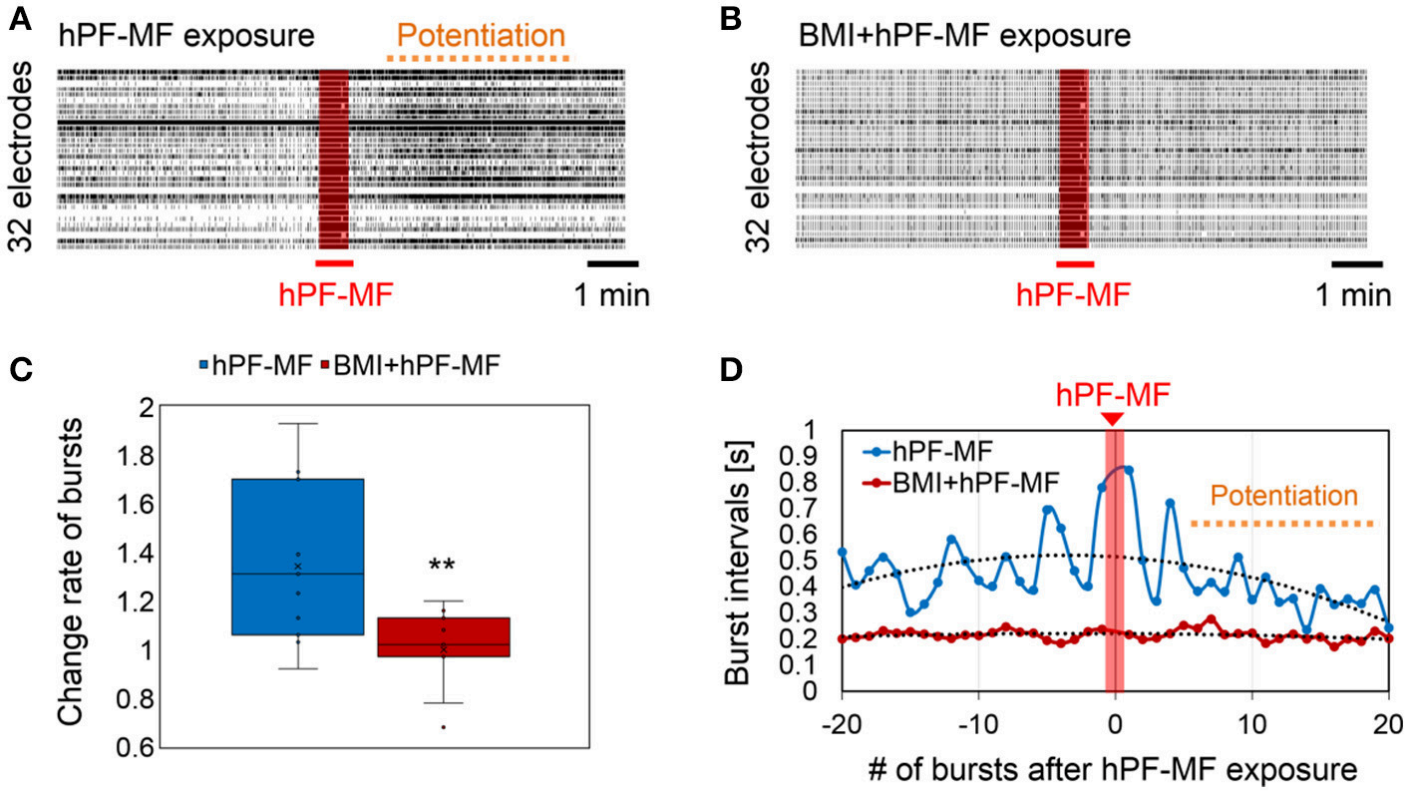
Change in hPF-MF exposure-related neuronal modulation after inhibition of the GABA receptor by BMI. **(A)** Comparison of raster plot data of neuronal activity **(A)** before and **(B)** after BMI application. Potentiation of neuronal activity after the 400 mT hPF-MF exposure disappeared after BMI. **(C)** Comparison of the change rate of synchronized bursting activity before and after BMI application. Increase in bursting activity after 400 mT hPF-MF exposure was suppressed after BMI. **(D)** Comparison of the time intervals of synchronized bursting activity before and after 400 mT exposure before (hPF-MF group) and after BMI application (BMI + hPF-MF group) (before and after 20 bursts). In the hPF-MF group, the time intervals of synchronized activities varied, whereas, these fluctuations were not observed in the BMI + hPF-MF group. Furthermore, the bursting intervals in the Normal group after 400 mT hPF-MF exposure tended to approach the bursting intervals in the BMI + hPF-MF group. *N* = 10 independent MEA dishes, ± SD, ^**^P < 0.0001.

As preparation, we performed 400 mT hPF-MF exposure on the cultured neuronal network and confirmed that the samples for the pharmacological experiments have the ability of the stimulus response (Figure 5A). When the stimulus response was evaluated again after application of BMI to the same sample, it was found that the enhancement of synchronized bursting activity due to 400 mT hPF-MF exposure had disappeared (Figures 5B,C). On the other hand, the time intervals of bursting activities tended to be shorter and stabilized with BMI application. The comparison between the 400 mT hPF-MF exposure without drug application (hPF-MF) and with BMI application (BMI + hPF-MF) group showed that the time variation of the bursting interval of the hPF-MF group tended to approach that of the BMI + hPF-MF group (Figure 5D). Based on the results, it was shown that 400 mT hPF-MF exposure was involved in the inhibition of inhibitory input through the GABA receptor.

### Modulation of pacemaker-like neuronal activity after hPF-MF exposure

**Figure 6:**
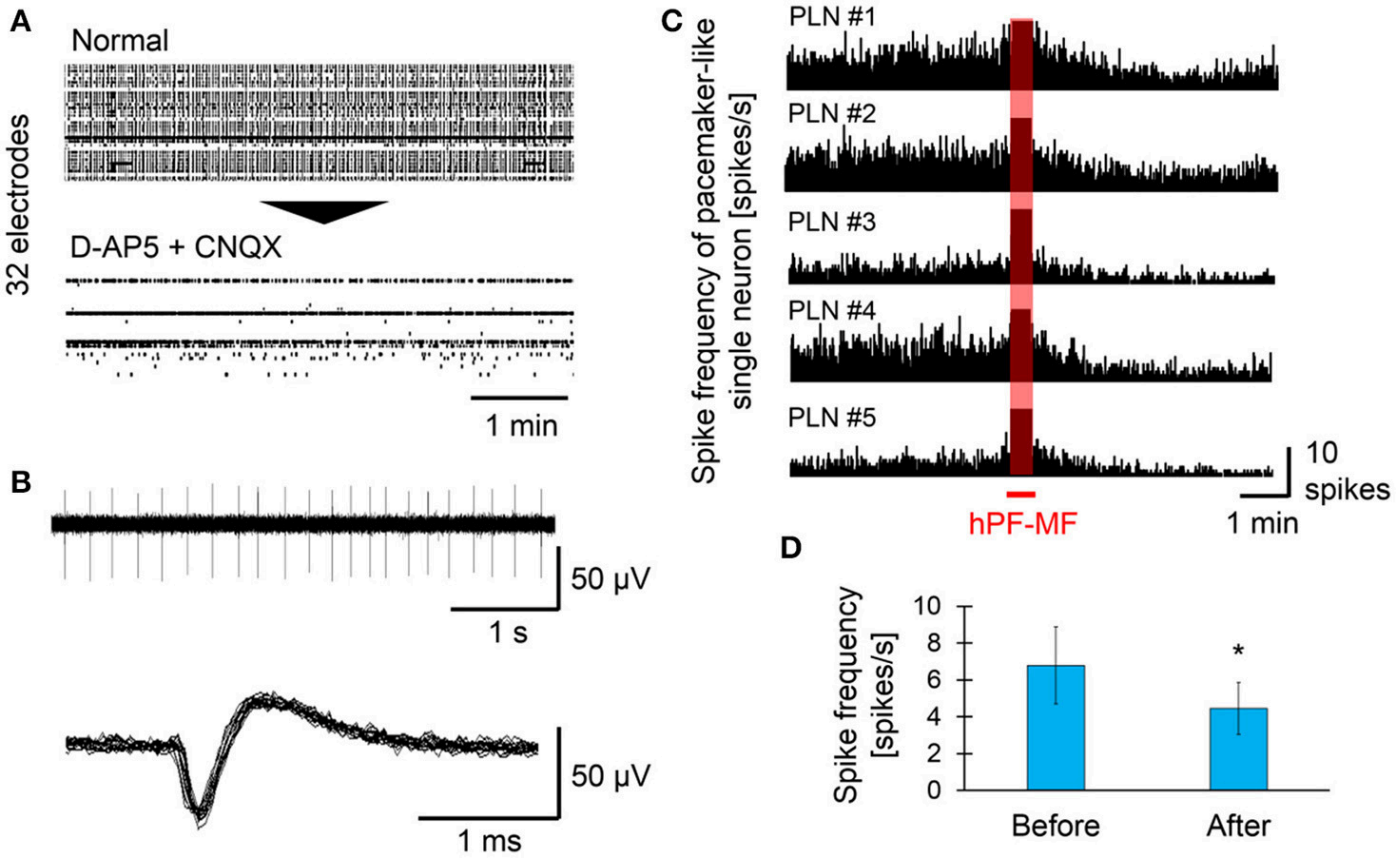
Effect of hPF-MF exposure on the autonomous activity of pacemaker-like neurons. **(A)** Screening of autonomous activity using MEAs and the pharmacological method. Even after D-AP5 and CNQX application, autonomous activity was detected in a few electrodes. **(B)** Detection of single pacemaker-like neuronal activity. Spontaneous activity with a frequency of 4–10 Hz, which is found in inhibitory neurons, was selected in some electrodes, then the effects of hPF-MF exposure on autonomous activity were evaluated. **(C)** Evaluation of the spike frequency after 400 mT hPF-MF exposure on the various pacemaker-like neurons. All autonomous activity detected from different cultures was reduced after 400 mT hPF-MF exposure. **(D)** Comparison of spike frequency before and after 400 mT hPF-MF exposure. *N* = 6 independent pacemaker-like neurons, ± SD, ^*^P < 0.005.

In this study, we attempted to select inhibitory neurons showing pacemaker-like activity based on extracellular potential recording using MEAs and pharmacological screening by D-AP5 and CNQX, and selected inhibitory pacemaker-like neurons exposed to hPF-MF. For selection of pacemaker-like neuronal activity after the addition of D-AP 5 and CNQX, matured samples with synchronized activity and inhibitory synapses were used. The raster plot data shown in Figure 6A indicate the changes of neuronal activity detected from the electrodes before and after the drug application. Synchronized activity detected from a wide range of MEAs disappeared after D-AP5 and CNQX application; however, autonomous spikes could be continuously measured on a few electrodes. Upon examining the characteristics of the firing pattern detected from the active electrode, the frequency of electrical activity of autonomously firing single neuronal cells was found to be 4–10 Hz (Figure 6B). In addition, the shape of the spike waveform showed that the rise time of the spike was less than 1 ms.

Subsequently, a few pharmacologically-selected cells were exposed to 400 mT hPF-MF to evaluate the effects of hPF-MF on pacemaker-like neuronal activity (Figure 6C). The spike frequency of pacemaker-like neuronal activity scarcely varied in different MEA dishes, and each cell was stable at a constant rhythm (4–10 spikes/s) before exposure to hPF-MF. However, it was found that spike frequency gradually decreased during about 1 min after the end of hPF-MF exposure. The number of spike firings before and after the MF exposure was 6.79 ± 2.09 and 4.45 ± 1.42 spikes/s, respectively, and the total number of spikes decreased by 0.6 times on average (Figure 6D). Furthermore, it was found that the time when the decrease of the autonomous activities in single pacemaker-like neurons was observed coincided with the timing related to the increase of the synchronized bursting activities shown in Figure 4. These results suggested that changes in neuronal activity due to hPF-MF exposure may be caused by inhibitory pacemaker-like neuronal activity and concomitant decrease in inhibitory input.

## Discussion

In this study, we aimed to detect the stimulus responses induced by high-intensity power frequency MF exposure in cultured neuronal network activity, and to clarify the threshold and mechanism of neuronal modulation by MF exposure.

Previous studies have reported values of 0.2–2°C as thresholds for thermal effects on neuronal activity (Adair et al., 1984; Michaelson and Elson, 1996; Saito et al., 2017). When evaluating our short-term hPF-MF exposure and neuronal activity recording scheme, we observed that the temperature in the culture medium was stabilized to under 0.2°C after MF exposure. However, a relatively large temperature rising of 10°C or more was observed from the room temperature (about 20°C) to the culture environment (about 37°C) at the stage of the experimental setup. For this reason, abrupt change in the medium temperature during this setup stage could not be avoided, and it was expected that it affected the neuronal activity immediately after setting. To address this issue, MF exposure experiments were started after temperature stabilization.

Our previous study reported the relationship between temperature elevation and modulation of synchronized neuronal network activity and indicated that (1) no influence on the number of synchronized neuronal network activity was seen in the range of 0.2–0.5°C, and (2) the temperature elevation over 0.5°C induces the “suppression” of neuronal network activity (Saito et al., 2017). Therefore, the “potentiation” of synchronized neuronal network activity observed in the 400 mT-exposed group was contrary to the thermal effects found in our previous results. On the other hand, we had to precisely evaluate the temperature elevation in the parts of the MEAs. To this end, another study that focused on the effects of radio frequency (RF) electromagnetic fields on cultured neuronal networks reported more stringent conditions (around 0.06°C) for temperature control for the MEAs (Moretti et al., 2013). In this study, we attempted recording the temperature elevation near the MEAs and confirmed temperature control maintained under 0.2°C during hPF-MF exposure. However, more detailed investigation of temperature elevation in single electrode resolution using numerical dosimetry is necessary for the precise evaluation of thermal effects.

In our first experiments, we examined the association of MF intensity with the modulation of cultured neuronal network activity. The results shown in Figure 3 revealed that clear responses were observed only in the 400 mT-exposed group; therefore, the threshold in this experiment was estimated to be in the range of 200–400 mT (rms). Based on theoretical estimation, it was indicated that the *E*-field induced in the neuronal networks in the culture medium ranged between 0.314 and 0.440 V/m at the 400 mT-exposed group. These values are about 19.8–27.7 times lower than 8.7 V/m, which is the estimated value of the stimulation threshold of nerve fibers in the central nervous system reported in the IEEE standard C95.6 (The Institute of Electrical and Electronics Engineers, 2002). Therefore, our experimental results showed that it is not through the direct excitation of the voltage-gated ion channel considered in the Spatially Extended Nonlinear Node (SENN) model (Reilly et al., 1985; Reilly, 1998). When compared to 0.133 V/m, which is an estimated threshold value of phosphene by synaptic modulation in the retina at 50 Hz (The Institute of Electrical and Electronics Engineers, 2002), the threshold obtained in our experiment was about 2.36–3.31 times higher. In addition, the *E*-field at 200 mT exposure was 0.157–0.220 V/m; therefore, neuronal modulation was not observed at the exposure intensity of 1.18–1.65 times that of the synaptic threshold value in the retina. From these results, it was expected that the modulatory threshold of synchronized bursting activity of the cultured neuronal network will be higher than the stimulation threshold estimated for the retina.

Previous studies have investigated the stimulus thresholds using power frequency (50/60 Hz) EF exposure, and reported that the *E*-field that modulated neuronal network activity was 0.1–0.2 V/m (Francis et al., 2003), 0.18–0.35 V/m (Deans et al., 2007), 0.75 V/m (Bawin et al., 1984, 1986), and 4–5 V/m (Jefferys, 1995; Saunders, 2003). Our results were 0.314–0.440 V/m, and closer to those reported by Deans et al. (Deans et al., 2007). Regarding such threshold differences, it is also important to consider physical aspects, such as differences in exposure properties (EF or MF, frequency component, waveform, and others). In order to clarify the details of these stimulation thresholds, both the numerical dosimetry in the culture medium to the MF exposure and the multi-scale simulation for evaluating the *E*-field in cells and tissues *in vitro* are required. In addition, regarding the threshold differences of phosphene by EF or MF exposure, it has been reported that the threshold value of MF-induced phosphine is about 3–10 times lower than that of EF (Reilly, 1998; Taki et al., 2003). In our study, we evaluated the *E*-field induced by 50-Hz sinusoidal MF exposure, and can be said that we produced findings that could be compared to those of the previous *in vitro* studies that investigated the threshold of EF exposure.

Evaluation at the synaptic level is necessary to investigate the mechanisms for the enhancement of the synchronized bursting activity observed in hPF-MF exposure. GABA receptors are involved in the suppression of neuronal excitement due to hyperpolarization of membrane potential via influx of Cl^−^ in the synaptic transmission of matured inhibitory neurons (Kandel et al., 2000). Interestingly, the pharmacological inhibition of the GABA receptor dissolved the response by hPF-MF exposure. At this time, the change in the time of bursting interval after hPF-MF exposure in normal conditions was similar to that of the bursting interval after inhibition of the GABA receptor. These results suggested that inhibitory synapses are essential for changes in synchronized bursting activity of neuronal networks, and that hPF-MF exposure suppresses the action of inhibitory synapses. Regarding the effect of MF exposure on the inhibitory synapse, plastic changes of inhibitory synapses by repetitive magnetic stimulation (rMS) have been reported by previous studies (Gramowski-Voß et al., 2015; Lenz et al., 2016). These studies reported that rMS was affected inhibitory synapses via the GABA receptor. Although these experimental methods were different from ours, the modulation of neuronal network activity by MF exposure was similar. In contrast, *in vivo* studies using rat brains reported that magnetic stimulation promotes GABA release and suppresses intracellular calcium influx of pyramidal neurons of the cortical L5 layer (Murphy et al., 2016). Comparing these *in vitro* and *in vivo* results, the mechanism of suppression via GABAergic neurons differs. In addition, it is well-known that neuronal plasticity is induced by long-term and repetitive electrical stimulation (Kandel et al., 2000). Here, the MEA-based extracellular recording method is suitable for non-invasive and long-term recording of neuronal network activity. Therefore, the investigation of neuronal modulation by long-term MF exposure is necessary for further study.

In order to investigate the neuronal modulation mechanism by MF exposure via inhibitory input, we focused on inhibitory pacemaker-like neurons. Previous studies (Frank and Mendelowitz, 2012) have shown that inhibitory neurons can be selected by pharmacological treatment; therefore, we detected pacemaker-like neuronal activity by combining this pharmacological method with an extracellular recording method using an MEA-based recording system. The spike frequency of the autonomous firing of single pacemaker-like neurons detected from a few electrodes was 4–10 Hz, which was confirmed with the highest reproducibility after D-AP5 and CNQX application. Here, it was reported that in the pacemaker neurons, which spontaneously fired at 4–10 Hz, the shape of the spike waveform showed that the rise time of the spike was less than 1 ms. Therefore, the detected autonomous spike frequencies and waveforms were similar to the activity of GABAergic, non-fast spiking, pacemaker neurons reported in previous studies (Serafin et al., 1996; Fischer et al., 1999; González-Burgos et al., 2005; Varga et al., 2008; Frank and Mendelowitz, 2012). Moreover, it was revealed that MF exposure reduces the frequency of autonomous activity of pacemaker-like neurons, and that this activity decline coincides with the timing of the potentiation of synchronized bursting activity. These results suggested that the depression of pacemaker-like neuronal activity and reduction of inhibitory input may lead to an increase of synchronized bursting activity. There have been several prior studies on the relationship between suppressive pacemaker-like neuronal activity and synchronized bursting activity (Wang, 2002; Compte et al., 2003). In particular, a previous model simulation showed increase of the frequency of synchronized activity by slightly decreasing the inhibitory input (Compte et al., 2003). These are useful findings to explain the mechanism of our experimental results.

Changes in firing frequency of GABAergic pacemaker-like neurons are observed at an intensity of *E*-field that is about 20–30 times lower than the threshold of neuronal cell membrane excitation (*E*-field (rms) was about 8.7 V/m) (The Institute of Electrical and Electronics Engineers, 2002). However, the modulatory mechanism of the spike frequency in GABAergic pacemaker-like neurons by MF exposure is not well understood. One possible mechanism is the influence via the hyperpolarization-activated cyclic nucleotide-gated (HCN) channel, which is present in GABAergic pacemaker neurons, opening with hyperpolarization, and involved in the generation of theta oscillation (Wang, 2002). Previous findings have shown that inhibition of HCN channels reduces the excitability of repressible pacemaker neurons (Varga et al., 2008). According to our results, the frequency of autonomous activity gradually decreased after hPF-MF exposure. Based on this result, further investigation is needed on the HCN channel inhibition mechanism and its threshold value due to MF exposure.

## Conclusion

We elucidated the modulatory effects of synchronized bursting activity and pacemaker-like autonomous activity in cultured neuronal networks using a hPF-MF exposure system combined with an *in vitro* multi-site recording method. Our results indicated that the synchronized activity was modulated by 400 mT (rms), 50 Hz, over 0.3 V/m exposure, and the suppression of pacemaker-like neuronal activity induced the potentiation of synchronized bursting activity. We expect these findings to be useful in the development of biological mechanisms for health risk assessment by MF exposure.

## Author contributions

AS performed most of the experiments and writing of the manuscript. MT performed the development of the experimental system. KM and YS performed the data analysis and technical suggestion of numerical dosimetry. YJ and SN supervised the experimental procedure and directed the research. All authors reviewed the manuscript and approved its submission.

### Conflict of interest statement

The authors declare that the research was conducted in the absence of any commercial or financial relationships that could be construed as a potential conflict of interest.

## References

[B1] Adair E. R. Adams B. V. Akel G. M. (1984). Minimal changes in hypothalamic temperature accompany microwave-induced alteration of thermoregulatory behavior. Bioelectromagnetics 5, 13–30. 10.1002/bem.2250050103 6712747

[B2] Azanza M. J. Calvo A. C. del Moral A. (2002). Evidence of synchronization of neuronal activity of molluscan brain ganglia induced by alternating 50 Hz applied magnetic field. Electromagn. Biol. Med. 21, 209–220. 10.1081/JBC-120015992

[B3] Azanza M. J. del Moral A. Calvo A. C. Pérez-Bruzón R. N. Junquera C. (2013). Synchronization dynamics induced on pairs of neurons under applied weak alternating magnetic fields. Comp. Biochem. Physiol. A Mol. Integr. Physiol. 166, 603–618. 10.1016/j.cbpa.2013.08.012 24012769

[B4] Balassa T. Varró P. Elek S. Drozdovszky O. Szemerszky R. Világi I.. (2013). Changes in synaptic efficacy in rat brain slices following extremely low-frequency magnetic field exposure at embryonic and early postnatal age. Int. J. Dev. Neurosci. 31, 724–730. 10.1016/j.ijdevneu.2013.08.004 24012627

[B5] Bawin S. M. Sheppard A. R. Mahoney M. D. Abu-Assal M. Adey W. R. (1986). Comparison between the effects of extra cellular direct and sinusoidal currents on the excitability in hippocampal slices. Brain Res. 362, 350–354. 10.1016/0006-8993(86)90461-0 3942883

[B6] Bawin S. M. Sheppard A. R. Mahoney M. D. Adey W. R. (1984). Influences of sinusoidal electric fields on excitability in the rat hippocampal slice. Brain Res. 323, 227–237. 10.1016/0006-8993(84)90293-2 6098340

[B7] Compte A. Sanchez-Vives M. V. McCormick D. A. Wang X. J. (2003). Cellular and network mechanisms of slow oscillatory activity (<1 Hz) and wave propagations in a cortical network model. J. Neurophysiol. 89, 2707–2725 10.1152/jn.00845.2002 12612051

[B8] D'Arsonval M. A. (1896). Dispositifs pour la mesure des courants alternatifs de toutes frequencies. CR Soc. Biol. 3, 450–451.

[B9] Deans J. K. Powell A. D. Jefferys J. G. (2007). Sensitivity of coherent oscillations in rat hippocampus to AC electric fields. J. Physiol. 583(Pt 2), 555–565. 10.1113/jphysiol.2007.137711 17599962 PMC2277040

[B10] Dimbylow P. (2005). Development of the female voxel phantom, NAOMI, and its application to calculations of induced current densities and electric fields from applied low frequency magnetic and electric fields. Phys. Med. Biol. 50, 1047–1070. 10.1088/0031-9155/50/6/002 15798308

[B11] Elmas O. Comlekci S. (2015). Investigation of effects of short-term exposure to 50 HZ magnetic field on central, peripheral, and autonomic nervous systems in rats. Bioelectromagnetics 36, 420–429. 10.1002/bem.21922 25974832

[B12] Fischer Y. Gähwiler B. H. Thompson S. M. (1999). Activation of intrinsic hippocampal theta oscillations by acetylcholine in rat septo-hippocampal cocultures. J. Physiol. 519(Pt 2), 405–413. 10.1111/j.1469-7793.1999.0405m.x 10457059 PMC2269511

[B13] Francis J. T. Gluckman B. J. Schiff S. J. (2003). Sensitivity of neurons to weak electric fields. J. Neurosci. 23, 7255–7261. 12917358 10.1523/JNEUROSCI.23-19-07255.2003 PMC6740448

[B14] Frank J. G. Mendelowitz D. (2012). Synaptic and intrinsic activation of GABAergic neurons in the cardiorespiratory brainstem network. PLoS ONE 7:e36459. 10.1371/journal.pone.0036459 22570717 PMC3343022

[B15] González-Burgos G. Krimer L. S. Povysheva N. V. Barrionuevo G. Lewis D. A. (2005). Functional properties of fast spiking interneurons and their synaptic connections with pyramidal cells in primate dorsolateral prefrontal cortex. J. Neurophysiol. 93, 942–953. 10.1152/jn.00787.2004 15385591

[B16] Gramowski-Voß A. Schwertle H. J. Pielka A. M. Schultz L. Steder A. Jügelt K.. (2015). Enhancement of cortical network activity *in vitro* and promotion of GABAergic neurogenesis by stimulation with an electromagnetic field with a 150 MHz carrier wave pulsed with an alternating 10 and 16 Hz modulation. Front. Neurol. 6:158. 10.3389/fneur.2015.00158 26236278 PMC4500930

[B17] Hirata A. Takano Y. Fujiwara O. Dovan T. Kavet R. (2011). An electric field induced in the retina and brain at threshold magnetic flux density causing magnetophosphenes. Phys. Med. Biol. 56, 4091–4101. 10.1088/0031-9155/56/13/022 21693787

[B18] International Commission on Non-Ionizing Radiation Protection (2010). Guidelines for limiting exposure to time-varying electric and magnetic fields (1 Hz to 100 kHz). Health Phys. 99, 818–836. 10.1097/HP.0b013e3181f06c86 21068601

[B19] Jefferys J. G. (1995). Nonsynaptic modulation of neuronal activity in the brain: electric currents and extracellular ions. Physiol. Rev. 75, 689–723. 10.1152/physrev.1995.75.4.689 7480159

[B20] Kandel E. R. Schwartz J. H. Jessell T. M. Siegelbaum S. A. Hudspeth A. J. (2000). Principles Neural Science, 5th Edn. New York, NY: McGraw-Hill Professional.

[B21] Legros A. Corbacio M. Beuter A. Modolo J. Goulet D. Prato F. S.. (2012). Neurophysiological and behavioral effects of a 60 Hz, 1,800 μT magnetic field in humans. Eur. J. Appl. Physiol. 112, 1751–1762. 10.1007/s00421-011-2130-x 21894451

[B22] Legros A. Modolo J. Brown S. Roberston J. Thomas A. W. (2015). Effects of a 60 Hz magnetic field exposure up to 3000 μT on human brain activation as measured by functional magnetic resonance imaging. PLoS ONE 10:e0132024. 10.1371/journal.pone.0132024 26214312 PMC4516358

[B23] Lenz M. Galanis C. Müller-Dahlhaus F. Opitz A. Wierenga C. J. Szabó G.. (2016). Repetitive magnetic stimulation induces plasticity of inhibitory synapses. Nat. Commun. 7:10020. 10.1038/ncomms10020 26743822 PMC4729863

[B24] Lövsund P. Oberg P. A. Nilsson S. E. (1979). Quantitative determination of thresholds of magnetophosphenes. Radio Sci. 14, 199–200. 10.1029/RS014i06Sp00199

[B25] Lövsund P. Oberg P. A. Nilsson S. E. Reuter T. (1980). Magnetophosphenes: a quantitative analysis of thresholds. Med. Biol. Eng. Comput. 18, 326–334. 10.1007/BF02443387 6968384

[B26] Manikonda P. K. Rajendra P. Devendranath D. Gunasekaran B. Channakeshava Aradhya R. S.. (2007). Influence of extremely low frequency magnetic fields on Ca2+ signaling and NMDA receptor functions in rat hippocampus. Neurosci. Lett. 413, 145–149. 10.1016/j.neulet.2006.11.048 17196332

[B27] Marino A. A. Nilsen E. Chesson A. L. Jr. Frilot C. (2004). Effect of low-frequency magnetic fields on brain electrical activity in human subjects. Clin. Neurophysiol. 115, 1195–1201. 10.1016/j.clinph.2003.12.023 15066545

[B28] Michaelson S. M. Elson E. C. (1996). Modulated fields and “window” effects, in Biological Effects of Electromagnetic Fields, eds Polk C. Postow E. (Boca Raton, FL: CRC Press), 435–533.

[B29] Moghadam M. K. Firoozabadi M. Janahmadi M. (2011). Effects of weak environmental magnetic fields on the spontaneous bioelectrical activity of snail neurons. J. Membr. Biol. 240, 63–71. 10.1007/s00232-011-9344-z 21249346

[B30] Moghadam M. K. Firoozabadi S. M. Janahmadi M. (2008). 50 Hz alternating extremely low frequency magnetic fields affect excitability, firing and action potential shape through interaction with ionic channels in snail neurones. Environmentalist 28, 341–347. 10.1007/s10669-007-9143-3

[B31] Moretti D. Garenne A. Haro E. Poulletier de Gannes F. Lagroye I. Lévêque P.. (2013). *In-vitro* exposure of neuronal networks to the GSM-1800 signal. Bioelectromagnetics 34, 571–578. 10.1002/bem.21805 23913345

[B32] Mukai Y. Shiina T. Jimbo Y. (2003). Continuous monitoring of developmental activity changes in cultured cortical networks. Electr. Eng. Jpn. 145, 28–37. 10.1002/eej.10216

[B33] Murphy S. C. Palmer L. M. Nyffeler T. Müri R. M. Larkum M. E. (2016). Transcranial magnetic stimulation (TMS) inhibits cortical dendrites. eLIFE 5:e13598. 10.7554/eLife.13598 26988796 PMC4811769

[B34] Nakanishi K. Kukita F. (1998). Functional synapses in synchronized bursting of neocortical neurons in culture. Brain Res. 795, 137–146. 10.1016/S0006-8993(98)00283-2 9622613

[B35] Nakasono S. Laramee C. Saiki H. McLeod K. J. (2003). Effect of power-frequency magnetic fields on genome-scale gene expression in *Saccharomyces Cerevisiae*. Radiat. Res. 160, 25–37. 10.1667/RR3006 12816520

[B36] Odawara A. Katoh H. Matsuda N. Suzuki I. (2016). Physiological maturation and drug responses of human induced pluripotent stem cell-derived cortical neuronal networks in long-term culture. Sci. Rep. 6:26181. 10.1038/srep26181 27188845 PMC4870631

[B37] Rauš B. S. Manojlović-Stojanoski M. Milošević V. Todorović D. Nikolić L. Petković B. (2016). Short- and long-term exposure to alternating magnetic field (50 Hz, 0.5 mT) affects rat pituitary ACTH cells: stereological study. Environ. Toxicol. 31, 461–468. 10.1002/tox.22059 25346405

[B38] Rauš S. Selaković V. Manojlović-Stojanoski M. Radenović L. Prolić Z. Janać B. (2013). Response of hippocampal neurons and glial cells to alternating magnetic field in gerbils submitted to global cerebral ischemia. Neurotox. Res. 23, 79–91. 10.1007/s12640-012-9333-8 22669750

[B39] Reilly J. P. (1998). Appied Bioelectricity. New York, NY: Springer-Verlag.

[B40] Reilly J. P. Freeman V. T. Larkin W. D. (1985). Sensory effects of transient electrical stimulation–evaluation with a neuroelectric model. IEEE Trans. Biomed. Eng. 32, 1001–1011. 10.1109/TBME.1985.325509 4077078

[B41] Saito A. Takahashi M. Jimbo Y. Nakasono S. (2017). Non-conductive and miniature fiber-optic imaging system for real-time detection of neuronal activity in time-varying electromagnetic fields. Biosens. Bioelectron. 87, 786–793. 10.1016/j.bios.2016.09.024 27649336

[B42] Saito A. Takayama Y. Moriguchi H. Kotani K. Jimbo Y. (2014). Induced current pharmacological split stimulation system for neuronal networks. IEEE Trans. Biomed. Eng. 61, 463–472. 10.1109/TBME.2013.2281079 24108746

[B43] Salunke B. P. Umathe S. N. Chavan J. G. (2014). Involvement of NMDA receptor in low-frequency magnetic field-induced anxiety in mice. Electromagn. Biol. Med. 33, 312–326. 10.3109/15368378.2013.839453 24131395

[B44] Saunders R. D. (2003). Rapporteur report: weak field interactions in the central nervous system. Radiat. Prot. Dosimetry 106, 357–361. 10.1093/oxfordjournals.rpd.a006372 14690279

[B45] Saunders R. D. Jefferys J. G. (2007). A neurobiological basis for ELF guidelines. Health Phys. 92, 596–603. 10.1097/01.HP.0000257856.83294.3e 17495661

[B46] Serafin M. Williams S. Khateb A. Fort P. Mühlethaler M. (1996). Rhythmic firing of medial septum non-cholinergic neurons. Neuroscience 75, 671–675. 10.1016/0306-4522(96)00349-1 8951863

[B47] Souques M. Plante M. Ostiguy G. Goulet D. Deschamps F. Mezei G. (2014). Anecdotal report of magnetophosphene perception in 50 mT 20, 50 and 60 Hz magnetic fields. Radioprotection 49, 69–71. 10.1051/radiopro/2013088

[B48] Steriade M. Amzica F. Nuñez A. (1993). Cholinergic and noradrenergic modulation of the slow (approximately 0.3 Hz) oscillation in neocortical cells. J. Neurophysiol. 70, 1385–1400. 8283204 10.1152/jn.1993.70.4.1385

[B49] Szemerszky R. Zelena D. Barna I. Bardos G. (2010). Stress-related endocrinological and psychopathological effects of short- and long-term 50Hz electromagnetic field exposure in rats. Brain Res. Bull. 81, 92–99. 10.1016/j.brainresbull.2009.10.015 19883742

[B50] Takahashi M. Saito A. Jimbo Y. Nakasono S. (2017). Evaluation of the effects of power-frequency magnetic fields on the electrical activity of cardiomyocytes differentiated from human induced pluripotent stem cells. J. Toxicol. Sci. 42, 223–231. 10.2131/jts.42.223 28321048

[B51] Takayama Y. Moriguchi H. Kotani K. Jimbo Y. (2009). Spontaneous calcium transients in cultured cortical networks during development. IEEE Trans. Biomed. Eng. 56, 2949–2956. 10.1109/TBME.2009.2028419 19695995

[B52] Taki M. Suzuki Y. Wake K. (2003). Dosimetry considerations in the head and retina for extremely low frequency electric fields. Radiat. Prot. Dosimetry 106, 349–356. 10.1093/oxfordjournals.rpd.a006371 14690278

[B53] The Institute of Electrical and Electronics Engineers (2002). IEEE Standard for safety levels with respect to human exposure to electromagnetic fields, 0–3 kHz. IEEE Std C95. 6. 10.1109/IEEESTD.2002.94143

[B54] Varga V. Hangya B. Kránitz K. Ludányi A. Zemankovics R. Katona I.. (2008). The presence of pacemaker HCN channels identifies theta rhythmic GABAergic neurons in the medial septum. J. Physiol. 586, 3893–3915. 10.1113/jphysiol.2008.155242 18565991 PMC2538919

[B55] Varró P. Szemerszky R. Bárdos G. Világi I. (2009). Changes in synaptic efficacy and seizure susceptibility in rat brain slices following extremely low-frequency electromagnetic field exposure. Bioelectromagnetics 30, 631–640. 10.1002/bem.20517 19572331

[B56] Wang X. J. (2002). Pacemaker neurons for the theta rhythm and their synchronization in the septohippocampal reciprocal loop. J. Neurophysiol. 87, 889–900. 10.1152/jn.00135.2001 11826054

[B57] World Health Organization (2007). Extremely Low Frequency Fields. Environmental Health Criteria. Geneva: WHO, 238.

[B58] Yang G. Ren Z. Mei Y. A. (2015). Exposure to 50 Hz magnetic field modulates GABA currents in cerebellar granule neurons through an EP receptor-mediated PKC pathway. J. Cell. Mol. Med. 19, 2413–2422. 10.1111/jcmm.12626 26176998 PMC4594682

